# Impact of a clinical encounter time protection program on pain management: a retrospective difference-in-difference study

**DOI:** 10.1093/intqhc/mzaf114

**Published:** 2025-10-30

**Authors:** Clement P Buclin, Denis Mongin, Nils Bürgisser, Amandine Berner, Pauline Darbellay Farhoumand, Jean-Luc Reny, Thomas Agoritsas, Delphine S Courvoisier

**Affiliations:** Division of General Internal Medicine, University Hospitals of Geneva, Geneva, Switzerland; Faculty of Medicine, University of Geneva, Geneva, Switzerland; Faculty of Medicine, University of Geneva, Geneva, Switzerland; Division of General Internal Medicine, University Hospitals of Geneva, Geneva, Switzerland; Division of General Internal Medicine, University Hospitals of Geneva, Geneva, Switzerland; Division of General Internal Medicine, University Hospitals of Geneva, Geneva, Switzerland; Division of General Internal Medicine, University Hospitals of Geneva, Geneva, Switzerland; Faculty of Medicine, University of Geneva, Geneva, Switzerland; Division of General Internal Medicine, University Hospitals of Geneva, Geneva, Switzerland; Faculty of Medicine, University of Geneva, Geneva, Switzerland; Department of Health Research Methods, Evidence, and Impact, McMaster University, Hamilton, Ontario, Canada; MAGIC Evidence Ecosystem Foundation, Oslo, Norway; Faculty of Medicine, University of Geneva, Geneva, Switzerland; Division of Quality of Care, University Hospitals of Geneva, Geneva, Switzerland

**Keywords:** health care quality, access, evaluation, quality indicators, health care, implementation science

## Abstract

**Background:**

Patient-centred care is a cornerstone for healthcare quality, leading many hospitals to implement programs that prioritize patient, even within time-constrained clinical environments. Effects of these programs are poorly evaluated.

**Methods:**

This retrospective observational cohort study aims to evaluate the impact of a patient-centred initiative aimed at protecting clinical encounter time by enhancing communication, fostering shared decision-making, and minimizing care interruptions on the quality of pain management in hospitalized patients. Electronic health record data and a difference-in-differences estimator were used to examine changes in quality of pain management outcomes in over 15 000 patients across 80 000 hospital stays ranging from 2018 to 2022 in the University Hospitals of Geneva, Switzerland.

Pain management quality was evaluated using timeliness of pain relief delivery, adequacy of pain documentation, and patient satisfaction with pain management. Data were compared in units who implemented the programs compared to matched control units to evaluate both the immediate and sustained effects of the program, accounting for potential selection and contamination biases.

**Results:**

The results demonstrated significant and sustained improvements in pain management quality in intervention units. Rates of timely painkiller administration increased significantly more in intervention units compared to control units (OR: 1.46, 95% CI: [1.37, 1.56]), with effects persisting over time. Pain documentation also showed significant improvement in intervention units (OR: 1.47, 95% CI: [1.15, 1.88]), although a minor initial decline was observed, likely due to temporary staffing disruptions during implementation. However, there was no significant improvement in patient-reported satisfaction with pain management, which may reflect limitations in survey sensitivity and response biases given the 53.3% response rate.

**Conclusion:**

This patient-centred program effectively improved objective measures of pain management quality in a tertiary hospital setting. However, further research is needed to assess its impact on patient experience, as well as healthcare professional engagement and satisfaction. Insights from future studies could guide the development of similar patient-centred initiatives that aim to balance efficiency with high-quality patient care.

## Introduction

Since the publication of the seminal article *Crossing the Quality Chasm* in 2001 [1], patient-centred care has been recognized as a key determinant of quality of care. This recognition has led many hospitals to reorganize their priorities and implement institutional programs aiming at placing inpatients at the centre of care [2–4]. Given the economic constraint on healthcare systems, most of these programs do not aim to increase the time allocated for clinical encounter directly. Instead, they focus on creating conditions that optimize the available time, fostering careful care and unhurried conversations between patients and healthcare professionals [5, 6]. However, our recent systematic review revealed that many of these programs are poorly evaluated or not evaluated at all [7].

At the University Hospitals of Geneva, a patient-centred program called ‘Plus de temps auprès des patients’, meaning ‘More Time at Patients’ side’ (MTP), was launched in 2017 [4]. This program introduced modular interventions for hospital units with three main goals: (i) to protect and optimize clinical encounter time between healthcare providers and patients; (ii) foster shared medical decisions; and (iii) improve interprofessional communication.

Despite its implementation in over 70 units, no formal ­evaluation of the program has been conducted. It is anticipated to improve the quality of clinical encounter time through enhanced communication, shared decision-making, and reduced interruptions.

Pain management is a frequent and critical component of inpatient care, with up to 84% of patients reporting pain during hospitalization [8]. Several aspects of pain management can be improved by optimizing interaction between patients and the healthcare teams. These include accurate documentation of pain, timely delivery of analgesics, and patient satisfaction with the overall pain management process. Timely care is particularly crucial in pain management, as delayed analgesia have been associated with longer length of stay and an increased risk of developing chronic pain [9, 10].

In this study, we assessed the impact of the MTP program on these three facets of pain management—timeliness of pain killer delivery, accurate pain documentation, and patient satisfaction with pain management—and their evolution over time, using a retrospective difference-in-difference design.

## Methods

To assess the impact of the MTP program on pain management, we evaluated the time evolution of key aspects of pain management. To mitigate potential confounding factors such as other institutional initiatives to improve pain management or national efforts to enhance pain management, we compared the time evolution of these outcomes and their changes after program implementation between units that implemented the MTP program and matched control units, where a fictional implementation date was assigned.

### Setting

The University Hospitals of Geneva (HUG) is a 2000-bed tertiary university hospital located in Geneva, Switzerland, which manages around 60 000 inpatients’ stays and 1.2 million outpatients’ visits annually.

### Intervention

**Figure 1:**
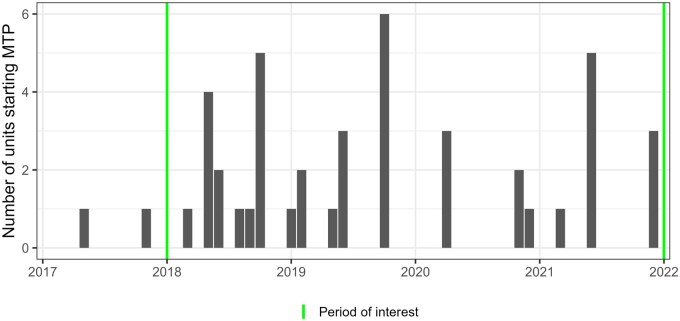
Implementation of the “More Time at Patients’ side” Program (MTP) in clinical units over time

The program called ‘Plus de temps auprès des patients’, meaning ‘More Time at Patients’ side’ (MTP), was launched in 2017 at HUG with the aim of simplifying clinical and administrative processes using tools from Lean Management and Design Thinking [11]. This program offers a toolbox of over 50 different interventions that each unit can select and adapt to their local context. These interventions encompass a comprehensive reorganization of care, including structured communication tools, time protection measures, care coordination methods, standardized operating procedures for frequent cares, role definitions for each professional group, checklists for essential materials needed for daily care, mandatory documentation processes, quality indicators with daily feedback, and continuous improvements processes. A full list of the MTP interventions is provided in Supplementary Materials (Supplementary Table S2). In each unit implementing MTP, a collaborative taskforce was formed, led by MTP experts and representatives from the units’ professional teams. Due to its emphasis on local adaptation, MTP was gradually implemented in over 70 units since its launch in 2017 (Fig. 1).

### Inclusion criteria

To ensure sufficient pre- and post-intervention data, we included units that implemented the MTP program (i.e. intervention units) between 2018 and 2021. Potential matched control units were identified as those that had not implemented MTP as of 21 December 2021. Pediatrics, psychiatry, intensive care, and intermediate care units were excluded from the control units (see matching details below). To minimize selection bias due to limited data availability at extreme time intervals from MTP implementation dates, the analysis window was restricted to a period of 1 year before to 2 years after implementation.

### Outcomes

#### Timely pain management

**Figure 2:**
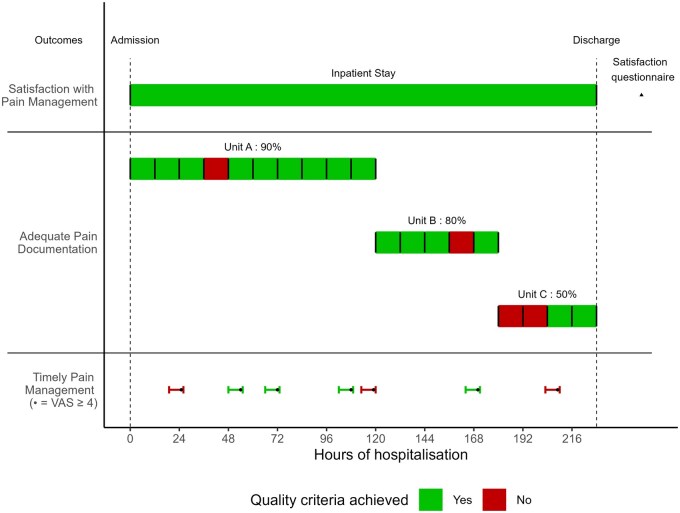
Chosen outcomes and their corresponding statistical units illustrated for a fictitious example. In this example, a fictitious patient is discharged after 9 days (216 hr) of hospitalization. In their post-discharge questionnaire, they declared being satisfied with pain management over their general hospital stay. During their stay, they transitioned through 3 units. In each unit, a percentage of 12 hr segments in which at least 1 Visual Analog Sale (VAS) is documented is computed: 9/10 = 90% in Unit A, 4/5 = 80% in Unit B and 2/4 = 50% in Unit C. The documentation is considered adequate in a specific unit when ≥90% of the 12 hr segments are documented. Finally, during their whole stay, for every occurrence of a documented VAS ≥4, a time window of −6 hr to +1 hr is queried for the delivery of a painkiller. Pain VAS is considered as managed in a timely fashion if at least 1 painkiller was delivered in that time window

The quality of pain management was defined as the timely delivery of a painkiller whenever a patient’s pain Visual Analog Scale (VAS) was ≥4 out of 10, as documented in the Electronic Health Record (EHR). Recognizing that documentation may often be delayed, nurses may record pain management actions after attending to all patient needs in the unit or closer to the end of their shifts, ‘timely’ was defined as occurring within a maximum of 6 hr before, and up to 1 hr after a pain Visual Analog Scale (VAS) was documented in the EHR (Fig. 2). This outcome is then computed for each documented VAS score of ≥4, regardless of any previous value.

#### Adequate pain documentation

Pain documentation was considered adequate when ≥90% of each patient’s hospitalization in 12-hr segments included at least one documented VAS score. This outcome was calculated at the unit stay level, with each transfer to a new unit resetting the calculation (Fig. 2).

#### Patient satisfaction with pain management

Patient satisfaction was assessed as a patient-reported outcome (PROM) using a questionnaire completed by patients 10 to 14 days after discharge. Patients were classified as satisfied if they reported no pain or, if they reported pain, responded “Yes, definitely” to the question about staff efforts to help manage their pain (Fig. 2). This information was obtained from patient satisfaction surveys routinely sent to all patients throughout the year. This survey uses validated items from the short version of the Picker Patient Experience Questionnaire to investigate eight aspects of patient-centred care, including physical comfort [12, 13].

### Statistical method

A logistic regression with a triple interaction was performed to evaluate the impact of the MTP program. The interaction included the following: (i) the immediate effect of being post-implementation date (in both MTP and control units); (ii) a continuous time difference (in years) from and to the implementation date; and (iii) whether the unit was a MTP or a matched control unit. To ensure clarity, the sequential logic of the analysis is preserved by first describing the matching process, followed by a detailed description of the regression model.

#### Matching with control units

A propensity score matching approach was used to assign control units with similar baseline characteristics to each intervention unit. Further details on the propensity scores are available in Supplementary Materials (Supplementary Table S1).

#### Statistical model

The change in each outcome over time following MTP implementation was analysed using logistic regression with random effects per units. In this difference-in-difference analysis, the change in time evolution of the outcome was estimated through an interaction effect involving three variables: *(1) immediate effect—*a variable indicating whether the time considered was before or after the MTP implementation in both control and intervention units; *(2) Time—*a continuous variable representing the number of years relative to the date MTP implementation; (3) *MTP implementation*—a variable indicating whether the unit was a MTP intervention unit. To further adjust for potential confounders, the models included adjustments for the number of patients’ comorbidities, ranging from 1 to 20 or more and patients’ gender as reported in the EHR. The analysis was conducted on R (v4.4.1) using the *lme4* library for the mixed effect modelling [14].

## Results

### Matching

The inclusion criteria identified 38 intervention units and 54 potential control units. After matching by propensity score, 38 pairs of units were successfully created based on data from the 6 months prior to the MTP implementation (Supplementary Table S1).

### Included patients and their characteristics

Data extracted from the matched units included a total of 363 221 documented VAS scores of ≥4. At baseline (prior to MTP implementation), patients in both groups were comparable in age, first self-reported language, and nationality. However, patients in MTP units were more likely to be female and had fewer documented comorbidities (Table 1). There were 15 709 unique patients hospitalized across 80 089 distinct unit stays, for whom the quality of pain documentation was evaluated. Among these, 8411 patients completed the satisfaction questionnaires, corresponding to a 53.5% response rate. MTP units exhibited substantial variability in the selected interventions aimed at improving clinical encounters (Supplementary Table S3).

**Table 1.** Unit stays’ characteristics

	Control units stays	MTP units stays^b^	*P*-value	Missing (%)
***N* ^a^**	6297	9412		
**Female (*N* (%))**	3383 (53.7)	5261 (55.9)	0.008	0
**Age (median [IQR])**	72.0 [56.0, 83.0]	72.0 [53.0, 84.0]	0.012	0
**Comorbidities (median [IQR])**	11.0 [5.0, 15.0]	9.0 [4.0, 14.0]	<0.001	5.1
**Language spoken (*N* (%))**				
French	5237 (83.2)	7873 (83.6)	0.177	0.6
German/Italian/English	853 (13.5)	1285 (13.7)		
Other	169 (2.7)	202 (2.1)		
**Nationality (*N* (%))**				
Swiss	4028 (64.0)	6032 (64.1)	0.775	1.2
European	1650 (26.2)	2440 (25.9)		
Other	553 (8.8)	825 (8.8)		
**Hospital department (*N* (%))**				
Surgery	1622 (25.8)	3010 (32.0)	<0.001	
Medicine	2560 (40.7)	2527 (26.8)		
Neurology	35 (0.6)	465 (4.9)		
Readaptation	2080 (33.0)	3410 (36.2)		

a *N* represents the number of patients’ stays in the units during the 6 months prior to intervention that was used to match them.

b M*TP* stands for the “More Time at Patient’s Side” program (MTP).

### Effects on the outcomes

#### Effect of the adjustment’s variables

For all three outcomes, number of comorbidities and gender had small but statistically significant effects (Table 2). Patients with a higher number of comorbidities were more likely to receive timely pain management but were less likely to have consistently adequate pain documentation (≥90% of 12-hr period) and less likely to report satisfaction with their pain management. Men were less likely to receive timely painkillers but more likely to have adequate pain documentation and significantly more likely to report satisfaction with their pain management.

**Table 2.** Linear regression models for the three quality of pain management outcomes: Timely Pain Management, Adequate Pain Documentation, and Satisfaction with Pain Management in the “More Time at Patient’s side” (MTP) and control units

	Outcome^a^								
	Timely Pain Management			Adequate Pain Documentation			Satisfaction with Pain Management		
	OR	95%CI	p-value	OR	95%CI	*P*-value	OR	95%CI	*P*-value
**Adjustment variables**									
Number of comorbidities	1.02	[1.02, 1.02]	<0.001	0.97	[0.97, 0.98]	<0.001	0.95	[0.94, 0.96]	<0.001
Male gender	0.94	[0.92, 0.95]	<0.001	1.06	[1.02, 1.10]	0.002	1.40	[1.23, 1.60]	<0.001
**Control Units**	*ref*	*ref*	*ref*	*ref*	*ref*	*ref*	*ref*	*ref*	*ref*
Change in effect per year before implementation date	1.33	[1.23, 1.42]	<0.001	1.68	[1.40, 2.01]	<0.001	0.75	[0.35, 1.59]	0.451
Immediate effect at implementation date	1.14	[1.08, 1.19]	<0.001	0.85	[0.76, 0.95]	0.006	0.76	[0.47, 1.25]	0.279
Change in effect per year after implementation date	0.82	[0.76, 0.88]	<0.001	1.08	[0.89, 1.30]	0.450	1.63	[0.74, 3.58]	0.226
**MTP Units**	0.84	[0.80, 0.89]	<0.001	0.52	[0.46, 0.58]	<0.001	0.67	[0.38, 1.16]	0.152
Change in effect per year before implementation date	1.20	[1.13, 1.28]	<0.001	1.07	[0.92, 1.25]	0.384	0.71	[0.38, 1.30]	0.265
Immediate effect at implementation date	1.66	[1.59, 1.73]	<0.001	0.90	[0.82, 0.98]	0.017	1.29	[0.87, 1.93]	0.205
Change in effect per year after implementation date	0.96	[0.89, 1.02]	0.217	1.58	[1.35, 1.86]	<0.001	1.59	[0.84, 3.02]	0.157
**Effect of MTP units versus control units**									
Difference of change in effect per year before implementation date	0.91	[0.82, 1.00]	0.046	0.64	[0.51, 0.81]	<0.001	0.94	[0.36, 2.50]	0.907
Difference of immediate effect at implementation date	1.46	[1.37, 1.56]	<0.001	1.05	[0.91, 1.22]	0.481	1.70	[0.91, 3.18]	0.099
Difference of change in effect per year after implementation date	1.17	[1.05, 1.30]	0.003	1.47	[1.15, 1.88]	0.002	0.98	[0.35, 2.70]	0.964

a This table displays the odd ratios from our logistic regression models for all three outcomes. The models were run twice: once with the “More Time at Patient’s Side” (MTP) units as the reference category and once with the control units as the reference. This approach simplifies the triple interaction effect into separate effects for MTP units and control unit. The “Effect of MTP units versus control units” displays the multiplicative effect of MTP units compared to control units, representing the relative impact of MTP implementation on the outcomes. The “Implementation date” refers to the date of MTP implementation for the intervention units and the corresponding matched implementation date for control units. Changes in effect per year represent effects over time whereas immediate effect rows are the instantaneous effect of the implementation of the program on each outcome. Effects of time are displayed for both MTP and control units to fully display the triple interaction: Immediate implementation of the program, Control versus MTP units and Time.

#### Timely pain management

Timely pain management was associated with both the implementation of MTP and with time from implementation in both MTP and control units. There was an immediate increase in the likelihood of timely pain management in both unit types (MTP units: OR: 1.66, 95% CI: [1.59, 1.73]; control units: OR: 1.14, 95% CI: [1.08, 1.19]). However, this immediate effect was significantly larger in MTP units than in control units (OR: 1.46, 95% CI: [1.37, 1.56]).

**Figure 3:**
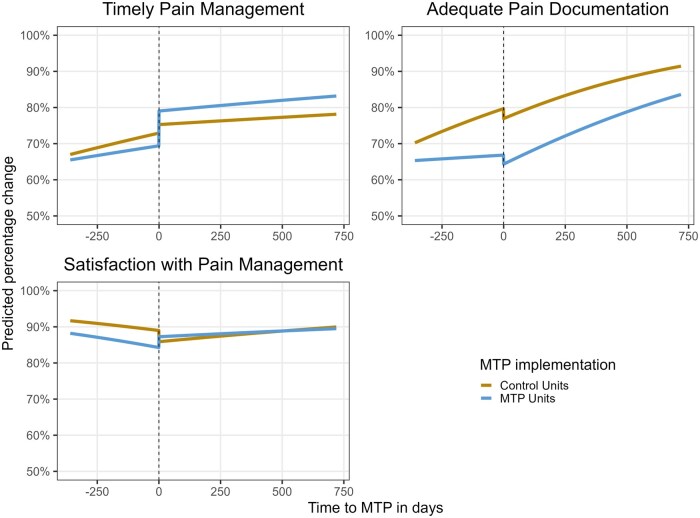
Predicted percentage change by the multivariable regression for each outcome. MTP stands for the “More Time at Patient’s Side” program (MTP)

In terms of time trends, both MTP and control units exhibited an improvement in timely pain management prior to MTP implementation (MTP units: OR: 1.20, 95% CI: [1.13, 1.28]; control units: OR per years: 1.33, 95% CI: [1.23; 1.42]). After implementation, the evolution of pain management quality diverged between the two groups (OR: 1.17, 95% CI: [1.05, 1.30]). In control units, the improvement trend reversed, showing a decline in the original improvement of quality over time (change of OR per year of 0.82, 95% CI: [0.76, 0.88]). In contrast, MTP units maintained a stable improvement trend (change of OR per year: 0.96, 95% CI: [0.89, 1.02]). This relative difference in time trends between MTP and control units after MTP implementation was ­statistically significant (OR: 1.17, 95% CI: [1.05, 1.30]) (Table 2 and Fig. 3).

#### Adequate pain documentation

The implementation of MTP had a similar immediate negative effect on pain documentation in both control and MTP units (control units: OR: 0.90, 95% CI: [0.82, 0.98]; MTP units: OR: 0.85, 95% CI: [0.76, 0.95]). After implementation, the rate of adequate documentation improved over time in both control and MTP units. However, the yearly improvement was significantly larger in MTP units (OR: 1.47, 95% CI: [1.15,1.88]) (Table 2 and Fig. 3).

#### Satisfaction with pain management

Satisfaction with pain management was not significantly impacted by the MTP program, either immediately after implementation or over time. However, a “catching-up” effect was observed in MTP units compared to control unit. Before implementation, satisfaction levels in MTP units were lower than those in control units, but this gap narrowed following implementation. A similar catching-up effect was also observed in the timely delivery of analgesics (Table 2 and Fig. 3).

## Discussion

### Statement of principal findings

In this difference-in-difference study of 15 000 patients, representing over 80 000 stays in hospital units of a large tertiary hospital, the implementation of a patient-centred program to protect clinical encounter time demonstrated both immediate and long-lasting significant effects on timely delivery of painkillers and adequate pain documentation. Before program implementation, pain management indicators were lower in intervention units, suggesting the program may have been introduced preferentially in units facing greater challenges in delivering high-quality care. At the implementation date, timely pain management improved in both intervention and control units, with a significantly larger effect in intervention units (OR: 1.46, 95% CI: [1.37, 1.56]). Over time, this difference widened, as the effect in intervention units remained stable over time, while it diminished in control units.

Adequate documentation rates were initially lower in intervention units, with no trend towards improvement before program implementation. After implementation, documentation improved over time in both intervention and control units, with a significantly larger effect observed in intervention units (OR: 1.47, 95% CI: [1.15, 1.88]). However, documentation rates immediately declined in both groups following implementation, with a smaller dip in adequate documentation observed in the intervention units. This initial decline may be attributed to temporary disorganization caused by the implementation process itself. During program implementation, healthcare professionals in targeted units were removed from direct clinical duties for up to a full day to receive training on the new strategies and care processes. This may have temporarily understaffed the units or required staffing with professionals from other units (e.g. control units) who were unfamiliar with the unit’s documentation practices, leading to a short-term negative impact on documentation rates [15].

The significant, albeit small, immediate effect observed in control units for timely pain management and adequate pain documentation may indicate a contamination effect [16, 17], consistent with findings from similarly designed studies [18, 19]. Several factors are likely to have contributed to this spillover. First, physicians and nurses frequently rotate between units, often within the same department, which were intentionally aligned in intervention and control units for this study. Additionally, the intervention program was widely publicized on the hospital intranet, potentially facilitating the dissemination of its strategies into control units. While this spillover effect represents a form of contamination in the study’s intervention, it is a welcomed and beneficial side-effect. It suggests that local investments in a unit can have broader, long-lasting positive impacts, extending beyond the unit itself to benefit the whole institution.

This study did not find a significant effect of the MTP program on patient satisfaction with pain management, although the observed changes paralleled those of timely painkiller administration: intervention units initially scored lower than control units but appeared to catch up following program implementation. This may be attributed to the design of patient satisfaction surveys, which emphasize high test–retest reliability over sensitivity to detect changes in patient care. Another plausible explanation is selection bias, particularly given that satisfaction is closely associated with individual patient factors such as cultural, psychological, and social background [20–22] and considering the relatively low response rate (53.3%) [23]. Changes in adequate pain documentation and timely delivery of painkillers might also not have translated into satisfaction with pain management due to recall bias as the questionnaire was sent two weeks after the discharge. Finally, originally very high satisfaction rates of around 90% leave limited room for measurable improvement and this outcome might suffer from a ceiling effect [24].

### Interpretation within the context of the wider literature

The MTP program introduced multiple interventions targeting diverse aspects of care, including improvements in communication, protection of patient encounter time, and standardization of roles and practices. However, there is a notable lack of prior literature examining their collective impact on pain management. Existing research has primarily focused on individual measures. For instance, efforts to enhance communication [25] or implement bedside handovers [26] have shown limited impact on pain management quality. In contrast, institutional recommendations for documentation [27] and patient-centred empowerment initiatives [28] have been associated with improved pain outcomes [29].

### Strengths and limitations

The main strength of this study lies in its large and representative evaluation of an institutional patient-centred program designed to protect clinical encounter time. By including all patients without exclusions, the study minimizes the risk of selection bias. Additionally, a rigorous methodology was employed to reduce confounding. The use of multiple indicators of pain management quality further strengthens the study by enabling a comprehensive evaluation of the program’s impact. However, this study also has several limitations. First, the primary outcome—timely pain management—is not a validated quality-of-care indicator and thus was developed specifically for this study. This indicator, like many others, has inherent limitations, such as not accounting for non-pharmacological pain management tools or physicians’ decision-making processes, which often relies on previous VAS values to guide subsequent treatment. Nonetheless, given the documented lack of validated quality indicators for acute pain management [30], developing this outcome allowed for the assessment of processes more closely aligned with patient needs, rather than focusing solely on documentation. Second and due to the retrospective design, the study lacks outcomes centred on healthcare professionals, despite the critical role of local champions in sustaining quality improvement initiatives over time [31, 32]. Third, the satisfaction with pain management outcome was based on a single item of a satisfaction survey completed up to 2 weeks after discharge and with a 53.3% response rate which may introduce recall bias and limit the reliability of the patient experience data. Finally, the program was implemented across diverse contexts with varying baseline standards of care, making it difficult to isolate which specific program elements contributed to the observed effects or to assess how contextual factors facilitated or hindered its success.

### Implications for policy, practice, and research

In time-constrained clinical settings, this study’s results suggest that healthcare institutions should prioritize fostering care environments that enable meaningful interactions and careful care. Emphasizing interprofessional organization and collaboration may enhance the quality of available time and improve patient outcomes. Despite its limitations, this study’s focus on the implementation of a multifaceted patient-centred initiative without inclusion criteria for the patients reinforces the generalizability of our study by offering pragmatic evidence of effects of a patient-centred program aiming to protect clinical encounter time.

A closer examination of the program reveals that the five most widely adopted interventions focused on improving communication and coordination. These included patient-facing communication tools such as whiteboards and scripted morning greetings, interprofessional coordination mechanisms such as daily huddles, and the formalization of multidisciplinary rounds. Each of these interventions showed modest effectiveness in previous studies. As such, they may represent valuable starting points for institutions aiming to design or adapt similar programs to improve patient care through enhanced team function and communication.

From a policy perspective, the study highlights the importance of including prospective evaluation plans when designing institutional programs. Implementing indicators from the outset would allow for more robust evidence by allowing randomization of intervention and the collection of patients and healthcare workers’ outcomes. Institutions planning to implement similar programs should collaborate with researcher to develop evaluation frameworks, including patient-relevant and cost-effectiveness outcomes, thereby producing higher quality evidence to guide future resource allocation.

Given that the outcomes used included non-validated measures and a single item extracted from a survey with a relatively low response rate, future research should focus on developing validated patient-reported outcome measures that evaluate the quality of pain management directly at the point of care. Nevertheless, asking already-burdened healthcare professionals to collect additional indicators should always be a last resort. Thus, we support alternative solutions using already collected day-to-day clinical data, similar to the timely pain management we proposed in this article. However, they would require more validation in designated studies in order to facilitate benchmarking across institutions and enable the dissemination of effective interventions.

## Conclusions

In conclusion, this study evaluating an institutional, patient-centred program aimed at protecting clinical encounter time demonstrated that intervention units significantly outperformed control units, both immediately and at long term, in timely painkiller administration and adequate documentation rates. While these improvements were accompanied by a non-significant increase in patient satisfaction with pain management, the findings highlight the potential of such programs to enhance key aspects of care quality.

## Supplementary Material

mzaf114_Supplementary_Data

## Data Availability

The dataset of this study is available from the corresponding author upon reasonable request.
