# Supplementary material for: Impact of a clinical encounter time protection program on pain management: a retrospective difference-in-difference study
Source: Int J Qual Health Care. 2025 Oct 30;37(4):mzaf114. doi: 10.1093/intqhc/mzaf114 (PMC12602149; doi:10.1093/intqhc/mzaf114)
Supplement: mzaf114_Supplementary_Data [file mzaf114_supplementary_data.zip › MTP_retrospective_analysis_SM.docx]

# Propensity scores and matching:

Propensity scores were constructed using three variables: *(1) Unit mean pain level* was calculated as the mean of all VAS pain scores recorded in the unit during the six months prior to MTP implementation; *(2) Unit mean comorbidity number* was derived by averaging the number of ICD-10 diagnoses per patient recorded in the EHR at discharge for all patients in the unit during the six months prior the MTP implementation; *(3) Unit mean nursing care charge* was evaluated using an internal scoring system that attributes a specific number of minutes to each nursing activity performed with a patient. Using this system, the overall mean daily nursing workload was calculated for the six months prior to MTP implementation.

In addition to the propensity score match, an exact match was required at the department level, as surgical units were deemed too different in pain levels and management strategies to allow for cross-department comparisons. Departments were classified as either medical (internal medicine, rehabilitation and neurology) or surgical.

For MTP units, the index date was the date of MTP implementation. For control units, the index date was set as the MTP implementation date of the matched intervention unit. To ensure robust matching, 1,000 random order samples of the intervention units were generated. For each sample, matches were created sequentially, and the sum of absolute differences in propensity scores between all 38 matched pairs was calculated. The match with the smallest total sum of absolute differences in propensity scores was retained as the final match.

Table S1 Units’ matching

| **Intervention (MTP)** | | | | | | **Control (No MTP)** | | | | |
| --- | --- | --- | --- | --- | --- | --- | --- | --- | --- | --- |
| Pair | **Department** | **Pain** | **Charge** | **Comorbidity** | **N stays** | **Department** | **Pain** | **Charge** | **Comorbidity** | **N stays** |
| 1 | Medical | 0.8 | 7.63 | 9.96 | 381 | Medical | 0.98 | 14.71 | 15.75 | 177 |
| 2 | Surgical | 2.28 | 11.43 | 5.27 | 1018 | Surgical | 1.39 | 16.42 | 9.75 | 401 |
| 3 | Medical | 0.82 | 7.49 | 9.63 | 390 | Medical | 1.09 | 8.72 | 14.53 | 170 |
| 4 | Medical | 1.61 | 8.68 | 13.48 | 65 | Medical | 1.63 | 15.2 | 18.14 | 273 |
| 5 | Medical | 1.11 | 13.28 | 12.26 | 133 | Medical | 0.59 | 12.75 | 13.17 | 230 |
| 6 | Medical | 1 | 9.63 | 10.59 | 402 | Medical | 1.82 | 7.76 | 14.01 | 181 |
| 7 | Medical | 1.28 | 9.02 | 12.47 | 83 | Medical | 2.68 | 9.11 | 14.31 | 140 |
| 8 | Medical | 0.79 | 16.92 | 14.05 | 429 | Medical | 2.65 | 13.07 | 13.35 | 480 |
| 9 | Medical | 0.84 | 12.1 | 9.66 | 571 | Medical | 0.87 | 8.11 | 6.73 | 113 |
| 10 | Medical | 1 | 13.84 | 16.55 | 132 | Medical | 0.91 | 8.45 | 13.12 | 163 |
| 11 | Medical | 0.75 | 8.33 | 11.27 | 357 | Medical | 1.35 | 8.31 | 11.97 | 147 |
| 12 | Medical | 0.92 | 11.84 | 14.13 | 86 | Medical | 0.26 | 12.4 | 13.71 | 261 |
| 13 | Medical | 0.97 | 12.15 | 11.6 | 158 | Medical | 2.64 | 11.72 | 14.66 | 252 |
| 14 | Medical | 1.38 | 10.86 | 12.86 | 58 | Medical | 0.21 | 13.26 | 12.69 | 54 |
| 15 | Medical | 1.42 | 12.91 | 14.44 | 116 | Medical | 0.74 | 8.49 | 13.49 | 190 |
| 16 | Medical | 1.02 | 15.5 | 11.88 | 251 | Medical | 1.28 | 18.19 | 13.89 | 270 |
| 17 | Medical | 1.02 | 9.97 | 11.39 | 317 | Medical | 0.58 | 8.31 | 13.1 | 155 |
| 18 | Medical | 1.3 | 10 | 12.93 | 446 | Medical | 1.31 | 9.19 | 12.1 | 59 |
| 19 | Medical | 1.03 | 14.45 | 11.7 | 246 | Medical | 2.94 | 6.87 | 8.4 | 307 |
| 20 | Medical | 0.67 | 13.82 | 14.12 | 183 | Medical | 1.55 | 7 | 9.66 | 132 |
| 21 | Medical | 1.65 | 12.48 | 16.22 | 157 | Medical | 0.3 | 6.65 | 9.96 | 28 |
| 22 | Medical | 1.26 | 10.03 | 16.12 | 119 | Medical | 0.99 | 7.74 | 13.78 | 27 |
| 23 | Medical | 1.98 | 6.58 | 15 | 90 | Medical | 0.79 | 10.56 | 17.7 | 10 |
| 24 | Medical | 1.23 | 15.63 | 12.59 | 226 | Medical | 0.86 | 10.42 | 8.72 | 431 |
| 25 | Medical | 1.53 | 8.48 | 12.41 | 76 | Medical | 0.7 | 13.21 | 15.38 | 73 |
| 26 | Medical | 1.08 | 16.29 | 14.63 | 404 | Medical | 1.39 | 12.91 | 13.83 | 96 |
| 27 | Surgical | 1.73 | 8.7 | 11.7 | 413 | Surgical | 1.3 | 4.97 | 8.38 | 410 |
| 28 | Surgical | 2.58 | 11.35 | 5.18 | 887 | Surgical | 2.05 | 14.09 | 8.14 | 123 |
| 29 | Medical | 1.57 | 13.49 | 12.43 | 586 | Medical | 0.81 | 13.91 | 13.22 | 37 |
| 30 | Medical | 0.46 | 10.65 | 7.97 | 539 | Medical | 3.21 | 12.53 | 12.56 | 34 |
| 31 | Medical | 1.41 | 7.49 | 6.82 | 294 | Medical | 1.24 | 12.84 | 10.77 | 13 |
| 32 | Medical | 0.69 | 8.89 | 9.94 | 464 | Medical | 0.52 | 12.52 | 12.28 | 61 |
| 33 | Medical | 1.97 | 9.38 | 3.18 | 1116 | Medical | 0.89 | 16.73 | 12.61 | 984 |
| 34 | Medical | 0.9 | 16.33 | 14.19 | 385 | Medical | 0.48 | 8.34 | 7.37 | 568 |
| 35 | Medical | 1.85 | 13.65 | 14.17 | 151 | Medical | 0.7 | 8.29 | 8.47 | 376 |
| 36 | Surgical | 0.83 | 9.44 | 7.01 | 446 | Surgical | 1.43 | 10.34 | 8.46 | 756 |
| 37 | Surgical | 1.43 | 12.2 | 7.21 | 880 | Surgical | 1.14 | 12.29 | 7.16 | 823 |
| 38 | Surgical | 1.32 | 12.35 | 8.34 | 860 | Surgical | 1.1 | 12.39 | 7.89 | 868 |

Table S2 List of existing standards for the MTP program

| Standard name | Standard | Short description |
| --- | --- | --- |
| Communication 1 | 7P | Structured communication (7 steps system) followed by the first healthcare professional interacting with the patient. Performed twice a day. Allows transmission of important information to the patient and clarification of patients’ needs. |
|  | 4P | Safety visits organized with a structured communication (4 steps system). Planned during the coordination meetings and performed each time a healthcare professional enters the patient’s room. Allows to proactively meet the patient’s needs and transmit important information. |
| Communication 2 | Patient whiteboard | Whiteboard by the patients’ bed with fields adapted to the units’ context but with notably: the date, name of unit, names of healthcare professionals responsible for the care of the patient, and contextually relevant field aiming to answer frequent questions of patients. |
| Communication 3 | Care plan for the patient whiteboard | List of the 3 main medical problems the patient is being cared for. The problems are described using a patient-adapted vocabulary, serves as a topic list for the medical visit and is displayed on the patient whiteboard. |
| Communication 4 | Common life rules | Operating and life charter for families/patients, with schedules and useful information about the unit |
| Communication 5 | Information upon admission | Standardized list of information to transmit to patients upon admission |
| Protection 1 | Coordinator healthcare professional | Each day one healthcare professional takes up the role of the coordinator to protect clinical work from interruptions (ex: answering units’ phone, welcoming and helping to orient visitors…). This professional also coordinates and plans out the day’s non-clinical activities. When the workload of the unit is heavy, this professional also coordinates with the unit’s other healthcare professionals to alleviate some of their clinical tasks. |
| Coordination 1 | Coordination meetings | Regular 2 minutes meetings several times per day at a paper board of the healthcare professional duo working on a given sector. Allows anticipation, planification, prioritization and adjustment to better fit the clinical activity of the sector. |
| Coordination 2 | Huddle | Daily multidisciplinary meeting (twice a day) around a whiteboard developed for the unit, aimed at coordinating care, identifying stakeholders and sharing important information. Short-interval facilitation to improve team performance. |
| Coordination 3 | Morning doctor-nurse meeting | Meeting of the nurse and doctor at the start of the day to identify urgent situations or problems requiring a rapid response. |
| Coordination 4 | End-of-day doctor-nurse meeting | End-of-day meeting of the nurse and doctor to review patients' progress over the day and take immediate note of new medical orders. |
| Coordination 5 | Unit’s whiteboard | Unit’s whiteboard adapted to the unit’s context that displays the predicted admissions and discharges of the unit. Allows for a global vision of the unit’s patient workload. |
| Coordination 6 | Admissions | Definition of a standardized admissions management process, including: person responsible, patient eligibility, zone, etc. |
| Coordination 7 | Discharges | Definition of a standardized discharge management process, including: person responsible, patient destination, transport, etc. |
| Coordination 8 | Morning kickoff | Organization of the start of the day, including structured morning transmission, preparation of treatments and carts, and transfer of telephones. |
| Coordination 9 | Afternoon kickoff | Organization of the start of the afternoon, including afternoon transmission, huddle, coordination points and telephone transfer |
| Coordination 10 | Nursing transmission: morning-evening | Standardization of transmission between morning and evening nurses. |
| Coordination 11 | Nursing transmission at bedside | Standardization of transmission between morning and evening nurses. In partnership with the patient and at the bedside. |
| Coordination 12 | Nursing transmission: evening-night | Standardization of transmission between evening and night nurses. |
| Coordination 13 | Nursing assistant transmission:  morning-evening | Standardization of transmission between morning and evening nursing assistant. Done in the nursing office. |
| Coordination 14 | Nursing assistant and Nurse transmission: night-morning | Standardization of transmission between evening and morning nursing assistant and nurses. |
| Coordination 15 | Easier appointments booking | A methodological framework for booking examination appointments that limits the loss of information. |
| Coordination 16 | Day plan | Plan of a typical day with meetings and huddles at scheduled times. |
| Coordination 17 | Synopsis | Standardized operating procedure for the filling of unit synopsis on the electronic health record. |
| Coordination 18 | Huddle Flux | Multidisciplinary meeting to coordinate patient flow information, including admissions, discharges and isolation measures. |
| Coordination 19 | Pre-weekend Coordination | Meeting of the nurse and doctor prior to start of the weekend to identify medical problems to follow up during the weekend and anticipate at-risk situations. |
| Coordination 20 | Late Huddle | Additional daily multidisciplinary meeting at the end of the day (when the coordinator healthcare professional leaves) held around a whiteboard developed for the unit. The aim is to coordinate care, identify those involved and share important information. |
| Coordination 21 | Huddle: Specialty module | Additional module for huddles, adding a maintenance point specific to the unit (e.g. rehabilitation). |
| Coordination 22 | Patient day ready | Standardization and checklist of preparations to be made at the start of the day to ensure that patients are ready for appointments and therapies. |
| Coordination 23 | Call to the intern | Decision help for nursing staffs on situations requiring a call to the intern and communication tools to communicate the situation efficiently. |
| Coordination 24 | Transfers to surgery | Standardization and checklist of preparations prior to an elective transfer to surgery. |
| Standardised care 1 | Multidisciplinary visit | Standardized multidisciplinary medical check-up, adapted to the context of the unit, with check-in, patient time, and check-out. |
| Standardised care 2 | Specific care standardized operating procedure | Standardized operating procedure for a specific and frequent care in the unit, established with unit members. |
| Standardised care 3 | Specialty conference | Standardization of attendance and roles for weekly multidisciplinary conferences for patient management specific to the unit (e.g. social, palliative, rehabilitation...). |
| Standardised care 4 | Specialty visit | Standardized multidisciplinary medical visits for specialists working on the unit (generally at a lower frequency than the medical/health-care visit). |
| Standardised care 5 | Patient groups | Group therapeutic services in the units (rehabilitation, physiotherapy, musicotherapy, psychotherapy, ...) |
| Standardised care 6 | Nurse clinical assessments | Standardization of daily clinical nursing assessments. |
| Indicators 1 | Patient satisfaction | Daily collection of patient satisfaction with at least 3 levels ☺ 😐 ☹. |
| Indicators 2 | Healthcare worker satisfaction | Collection of employee satisfaction on a whiteboard to improve working conditions and interprofessionality. |
| Role 1 | Morning nurse role | Definition of unit-specific roles and tasks for the morning nurse. |
| Role 2 | Evening nurse role | Definition of unit-specific roles and tasks for the evening nurse. |
| Role 3 | Morning nursing assistant role | Definition of unit-specific roles and tasks for the morning nursing assistant. |
| Role 4 | Evening nursing assistant role | Definition of unit-specific roles and tasks for the evening nursing assistant. |
| Role 5 | Physiotherapist | Definition of unit-specific roles and tasks for the physiotherapist. |
| Role 6 | Head nurse | Definition of unit-specific roles and tasks for the head nurse. |
| Role 7 | Intern and clinical fellow | Definition of unit-specific roles and tasks for the junior and senior doctors. |
| Role 8 | Clinical fellow + attending | Definition of unit-specific roles and tasks for the attending senior doctors, notably acting as role model for the standards. |
| Role 9 | Case managers | Definition of unit-specific roles and tasks for case managers who are notably in charge of appointments for out-of-hospital patients, and post-hospital care (transfer to rehabilitation centers, nursing homes, etc.). |
| Role 10 | Human resources | Definition of unit-specific roles and tasks for the human resource representant. |
| Role 11 | Secretariat | Definition of secretarial roles, including: taking calls during the huddles or the visit, clarification of calls requiring interruption of the visit (e.g. urgent lab results). |
| Role 12 | Night nurse and nursing assistant role | Definition of unit-specific roles and tasks for the night nurses and nursing assistants. |
| Role 13 | In-unit catering staff | Definition of unit-specific roles and tasks for the in-unit catering staff. |
| Role 14 | Cleaning and hygiene staff. | Coordination of tasks during the day between care teams and cleaning and hygiene staff, to avoid scheduling conflicts. |
| Checklists 1 | Cart contents checklists | List of cart contents for the various professions, other than the specific cart for nurses and nursing assistants, and definition of roles for maintaining completeness. |
| Checklists 2 | In-room material checklists | List of equipment that must be present in the room to facilitate care or patient access. |
| Checklists 3 | In-room cabinet checklists | List of equipment to be kept in the in-room care cabinet |
| Checklists 4 | Specific care material checklists | List and defined locations for materials specific to frequent care in the unit. |
| Checklists 5 | 5S | Checklist of storage space contents. |
| Checklists 6 | Nursing assistant carts | Checklist of the contents of the nursing assistants’ cart, which must be ready to meet the day's needs and can be brought into the room for care. |
| Checklists 7 | Nurse carts | Checklist of the contents of the nurse carts which must be ready to meet the day’s needs and can be brought into the room for care. |
| Documentation 1 | Immediate documentation | Immediate, bedside documentation of vitals and medication dispensing for more reliable and complete documentation. |
| CONTINUOUS IMPROVEMENT 1 | Kaizen | Continuous improvement idea collection chart that is reviewed 1x/2 months to try to implement the proposed solutions. Accompanied by a team and a process for implementing the proposed ideas. |
| CONTINUOUS IMPROVEMENT 2 | Continuing education | Weekly continuing education meeting with a theme chosen and presented by the medical and nursing teams. |
